# Electrospun silk fibroin/poly (L-lactide-ε-caplacton) graft with platelet-rich growth factor for inducing smooth muscle cell growth and infiltration

**DOI:** 10.1093/rb/rbw026

**Published:** 2016-07-15

**Authors:** Anlin Yin, Gary L. Bowlin, Rifang Luo, Xingdong Zhang, Yunbing Wang, Xiumei Mo

**Affiliations:** ^1^National Engineering Research Center for Biomaterials, Sichuan University, Chengdu 610064, China;; ^2^Department of Biomedical Engineering, University of Memphis, Memphis, TN 38152, USA; ^3^College of Chemistry, Chemical Engineering and Biotechnology, Donghua University, Shanghai 201620, China

**Keywords:** vascular graft, PRGF, smooth muscle cell infiltration, electrospinning

## Abstract

The construction of a smooth muscle layer for blood vessel through electrospinning method plays a key role in vascular tissue engineering. However, smooth muscle cells (SMCs) penetration into the electrospun graft to form a smooth muscle layer is limited due to the dense packing of fibers and lack of inducing factors. In this paper, silk fibroin/poly (L-lactide-ε-caplacton) (SF/PLLA-CL) vascular graft loaded with platelet-rich growth factor (PRGF) was fabricated by electrospinning. The *in vitro* results showed that SMCs cultured in the graft grew fast, and the incorporation of PRGF could induce deeper SMCs infiltrating compared to the SF/PLLA-CL graft alone. Mechanical properties measurement showed that PRGF-incorporated graft had proper tensile stress, suture retention strength, burst pressure and compliance which could match the demand of native blood vessel. The success in the fabrication of PRGF-incorporated SF/PLLA-CL graft to induce fast SMCs growth and their strong penetration into graft has important application for tissue-engineered blood vessels.

## Introduction

Suitable blood vessel substitutes, especially small diameter (inner diameter ≤ 4 mm) vessels, are in the great need for coronary artery disease in clinical therapy [1]. So far, the autologous saphenous vein remains the first choice to replace diseased vascular tissue. However, it may not be available for some patients due to vascular disease, amputation and previous harvest. Moreover, the use of this kind of vein requires a secondary surgical procedure to obtain the vessel [2]. Currently, vascular grafts based on polyethylene terephthalate (Dacron), expanded polytetrafluoroethylene (ePTFE) and decellularized bovine ureters are used in clinics [3–5]. Dacron and ePTFE are synthesized polymeric materials those have limited success when used as small-diameter arterial substitutes whereas they have been successfully used as large-caliber arterial substitutes [2]. The decellularized tissues are increasingly attracting attention since these materials are naturally derived and inherently sustain cell behaviors [6, 7]. However, the batch-to-batch variations of allograft raw materials have hampered the application of decellularized tissues. In addition, the significant immune response may be caused by the xenografts in the recipient [8, 9]. Thus, there remains an urgent clinical demand for functional small diameter arterial graft preparation.

To construct a functional small diameter vascular graft, it is essential to mimic the *in vivo* environment. The vascular structure consists of three concentric layers, including intimae, media and adventitia [10–12]. Among these three layers, the media layer which is circumferentially oriented by smooth muscle cells (SMCs) plays an important role in maintaining elasticity, mechanical strength and vasoactive responsiveness of the blood vessels [1, 13, 14]. Therefore, fabricating a vascular graft which facilitates the SMCs to penetrate into the inner graft is a prerequisite, so as to construct an integral media layer for mimicking the function of smooth muscle layer. To date, electrospinning technology has become an attractive method, which can control the composition, structure and mechanical properties of fibrous scaffolds. Moreover, the drugs, growth factors and other biomolecular signaling agents could be combined together in the polymeric solution for electrospinning [2]. A variety of biodegradable polymers, including natural materials such as collagen [15], elastin [16], silk fibroin [2] and synthetics like poly(lactic acid) [17], polycaprolactone (PCL) [18] and poly (L-lactide-ε-caplacton) (PLLA-CL) [2, 15], have been investigated via electrospinning for fabrication of vascular scaffolds. Silk fibroin (SF) is a natural protein which has been widely used in tissue engineering due to its good biocompatibility and relatively low cost [19–21]. PLLA-CL is a biodegradable copolymer which possesses good mechanical properties, however, as a synthetic material, it lacks the natural integrin binding domains for proper cellular interactions [22]. In previous research, electrospun SF/PLLA-CL composite nanofibrous scaffolds had been fabricated in order to combine the advantage of both SF and PLLA-CL, and the results indicated that SF/PLLA-CL scaffold had the combined physical properties and biocompatibility [2, 20].

In recent years, platelet-rich plasma (PRP) therapy has attracted clinical interest due to the potential for stimulating tissue repair and regeneration. Platelet-rich growth factors (PRGF) [23], which is produced from PRP would stimulate chemotaxis and mitogenesis in SMCs [24]. PRGF therapy has gained a lot of attention for its good effect on treating surgery involved in ligament reconstruction [25], improving bone healing [26]. Moreover, PRGF therapy has also been used to repair tendon [27] and cartilage injuries [28].Yet, among those published PRGF studies, very few are referred to blood vessel restored by vascular grafts.

In this study, PLLA-CL, SF and PRGF are used to fabricate SF/PLLA-CL/PRGF vascular graft by electrospinning. PRGF-incorporation is introduced to promote effective SMCs growth and infiltration into the graft to construct a smooth muscle layer. The fabricated graft would be characterized to determine their potential application as a vascular scaffold/prosthetic. This study would explore cell growth and penetration into the graft by PRGF stimulation, and calculate the depth of cellular infiltration. Furthermore, mechanical properties of the graft would be evaluated in terms of tensile testing, suture retention, burst pressure and compliance.

## Materials and methods

### Preparation of PRGF

PRGF was obtained from fresh human whole blood according to previous procedure [23]. Briefly, blood from donors was initially centrifuged to get PRP and was then subjected to a freeze-thaw-freeze cycle in a −70°C freezer for cell lysis and activation. Finally, the frozen PRP was lyophilized for 24 h to create a dry PRGF powder, which was finely ground in a mortar and pestle before use.

### Silk fibroin extraction

SF was extracted from the cocoons of Bombyx mori silkworms (The Yarn Tree, NY, USA) following a published protocol [2]. Briefly, cocoons were cut into pieces and boiled in a 0.02 M Na_2_CO_3_ (Sigma-Aldrich) solution for 30 min to remove the sericin gum, followed by a thorough rinsing in deionized water (DI), and then were air-dried overnight in a fume hood. The SF was then dissolved in a LiBr (Fisher Scientific) solution at 60°C for 1 h, and dialyzed against DI for 3 days using 12 000–14 000 Molecular weight cut off dialysis tubing (Fisher Scientific). The SF solution was then frozen and lyophilized to provide pure SF for electrospinning.

### Electrospinning

PLLA-CL polymer (MW: 300 000 Da, LA to CL mole ratio at 50:50, Gunze Limited, Japan) and SF were dissolved in 1,1,1,3,3,3-hexafluoro-2-propanol (TCI America) at 120 mg/ml, respectively. PLLA-CL and SF solutions were blended at a volume ratio of 70:30. PRGF was then added in concentrations of 20 mg per milliliter of electrospinning solution. Before electrospinning, the blended solution was loaded into a 5 ml plastic Becton Dickinson syringe with a 16 G blunt tip needle and dispensed at a rate of 3 ml/h. The needle tip was subjected to +30 kV with an air gap distance of 20 cm between the needle and the grounded aluminum foil collector. To fabricate the tubular grafts, the collector was a solid cylindrical stainless mandrel with 4 mm in diameter. The deposited fibrous sheet or conduit was dried in a vacuum oven at room temperature for 7 days to remove any residual solvents.

### Morphology characterization of the graft

The morphology characterization was performed using scanning electron microscopy (SEM, JEOL JSM-5610LV, Japan). SEM micrographs were analyzed with a software Image-J (National Institutes of Health). The average fiber diameter was determined by measuring 50 randomly selected fibers in the SEM image. Calibration of the Image Tool software was achieved by using the scale bar on each image. Pore size was measured by determining distance based on both the most superficial fibers and by measuring from one fiber to the next closest fiber according to the early reports [29].

### Cellular analysis

In order to study the infiltration and proliferation of cells in grafts, human umbilical artery smooth muscle cells (HUASMCs, ScienCell) were seeded on the grafts. SMC medium (ScienCell) supplemented with 2% Fetal bovine serum (ScienCell), 1% Smooth muscle cell growth supplement [] (ScienCell) and 1% P/S (ScienCell) was used. Ten millimeter biopsy punch was taken from grafts to make specimens, and specimens were placed into 48-well plates, disinfected by soaking in 75% ethanol (Sigma) for 30 min, and washing with phosphate-buffered saline (PBS), then seeded with 100 μl of the cell suspension (cell concentration of 100 000 cells/ml), and placed in an incubator at 37°C and 5% CO_2_ for 1 or 2 weeks. The medium was refreshed every 2 days.

For cellular infiltration measurement, HUASMCs were cultured for 1, 7 and 14 days. After cellular interaction time periods, cells-graft cultures were first fixed using 10% buffered formalin for 24 h at 4°C, then embedded in Optimal Cutting Temperature (O.C.T) Compound (Tissue-Tek) and frozen at −80°C. Frozen samples were then sectioned at a thickness of 30 μm and stained with 4, 6-diamidino-2-phenylindole (DAPI, Invitrogen). All samples were imaged using a Nikon TE300 microscope. Ultraviolet light was used to capture DAPI stained nuclei. Cell depth was measured using Image-J software. Briefly, images were first imported, and calibrated according to the scale bar, and then measurements were taken from the edge of the scaffold to the center of the furthest cell nuclei in line with the measurement tool.

Cell viability on the different types of membranes at day 1, 4 and 7 was assessed by the cell counting kit-8 (CCK-8 Kit, Beijing Zoman Biotech, China). Briefly, the culture medium was replaced with 350 μl medium and 50 μl CCK-8 solution. They were incubated at 37°C for 3 h and then 100 μl supernatant was transferred to the 96-well plate. The absorbance was measured at 450 nm using a microtiter plate reader (Multiskan MK3, Thermo, USA). Six parallel experimental samples were used to assess the cell viability in this study.

After 1, 4 and 7 days culture *in vitro*, HUASMCs were observed by a staining procedure using fluorescein diacetate, and the living cells were observed by a confocal laser scanning microscope (CLSM, Leica TCSSP5, Germany).

### Uniaxial tensile testing

For tensile testing, specimens in a ‘dog bone’ shape were punched from electrospun mats (sample size: 2.75 mm wide at their narrowest point with a length of 7.5 mm) and were hydrated in PBS for 6 h before testing. Uniaxial tensile testing was performed on an MTS Bionix 200 testing system with a 100 N load cell (MTS Systems Corp.) and with an extension rate of 10.0 mm/min. Modulus, peak stress and strain at break were calculated using Test Works version 4 (software).

### Suture retention strength

Suture retention strength was measured with a rectangular test sample (10 mm in width/20 mm in length). Before testing, one end of the graft was clamped to one arm of the micro material testing machine (MMT-250N, Shimadzu Co., Japan). A loop of a 5-0 polyester suture (Shanghai Pudong Jinhuan Medical Products Co., Ltd, China) was placed 2 mm from the edge of the free end of the sample and clamped to the other arm which moved at a constant speed of 120 mm/min until failure. The suture retention strength was defined as the peak force obtained during the procedure (*n* = 6 for each graft type). All samples were kept hydrated throughout the testing protocols.

### Burst pressure

Burst strength testing of electrospun grafts was completed using a device designed in accordance with Section 8.3.3.3 of ANSI/AAMI VP20:1994.31,32 [2]. Tubes with 4 cm in length were hydrated in PBS for 6 h, fitted over 2.5 mm diameter nipples attached to the device, then a thin latex balloon (Party Like Crazy, Target) was inserted, and the balloon/scaffold was secured with 2-0 silk suture to the nipples. At last, pressurized air was introduced into the system with increased pressure at a rate of 5 mmHg/s until the tubes ruptured. Burst pressure (mmHg) were recorded when the structures ruptured.

### Dynamic compliance

Dynamic compliance was determined for tubular grafts with a length of 4 cm under simulated physiological conditions in accordance with Section 8.10 of ANSI/AAMI VP20:1994.32,33 [2]. Grafts were soaked in PBS for 6 h before testing. The specimens were tested in a bioreactor developed by Tissue Growth Technologies (Minnetonka, MN) filled with PBS. The bioreactor provided a cyclic (1 Hz, representing 60 beats per minute) pressure change to the inside of the graft at a pressure level of 120/80 mmHg systolic/diastolic. Prior to compliance measurements, all grafts were allowed to stress relax for 600 cycles. Internal pressure was measured with a pressure transducer capable of measuring dynamic pressure up to 200 ± 2 mmHg, while the external diameter of the graft was recorded with a laser micrometer system with an accuracy of ±0.001 mm. Compliance was calculated through recording of pressure and inner diameter as:

$$\%\text{ Compliance=}\frac{R_{{\text{P}}_{2}}-R_{{\text{P}}_{1}}}{R_{{\text{P}}_{1}}}\frac{1}{{\text{P}}_{2}-{\text{P}}_{1}}\times 10^{4}$$

while *R* is the internal radius, *P*_1_ is the lower internal pressure and *P*_2_ is the higher internal pressure.

### Statistics analysis

All the data were obtained at least in triplicate and expressed as means ± standard deviation (SD). One-way ANOVA at a significance of *P* < 0.05 was performed using Origin 8.0 (Origin Lab, USA).

## Results and discussion

### Graft characterization

**Figure 1.**
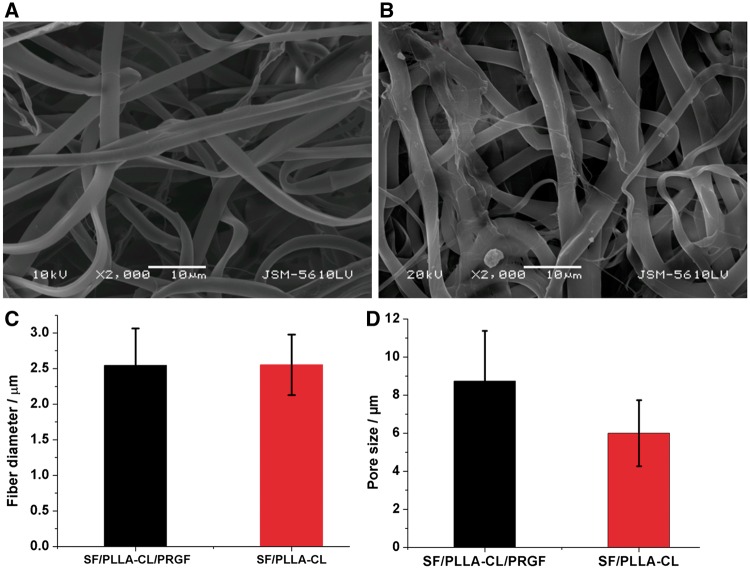
The SEM images of electrospun fibers and fiber diameters. **(A)** SF/PLLA-CL/PRGF. **(B)** SF/PLLA-CL. **(C)** Fiber diameter distributions of grafts. **(D)** Pore size of different grafts

The morphologies of SF/PLLA-CL/PRGF fibers collected by aluminum foil were observed under SEM and shown in Fig. 1A and B. The fibers appeared macroscopically smooth without any gross defects. From the micrographs, it was clear that with PRGF-incorporation, the fibers deposited looser than that without PRGF. While fiber diameter (2.5 μm) of this two grafts has no significant difference shown in Fig.1C. Figure 1D displayed that the SF/PLLA-CL/PRGF with the largest pore size (8.9 ± 2.5 μm) than that of SF/PLLA-CL with an evaluated value of 5.8 ± 1.7 μm, which probably contributed to deeper cellular infiltration.

### Cellular behavior

Graft for tissue engineering are typically designed to promote cell growth, infiltration, physiological functions and to maintain normal states of cell differentiation [30, 31]. Electrospun fibrous scaffolds can simulate the extracellular matrix structure of the native tissue in both structure and composition. Unfortunately, the dense packing of the fibrous network impedes cellular infiltration. As a vascular graft, SMCs penetration to construct a functional smooth muscle layer is a prerequisite. In addition, SMCs need to grow fast for secreting ECM, corporate to reestablish the media layer of vascular tissue.

#### Cellular infiltration

**Figure 2.**
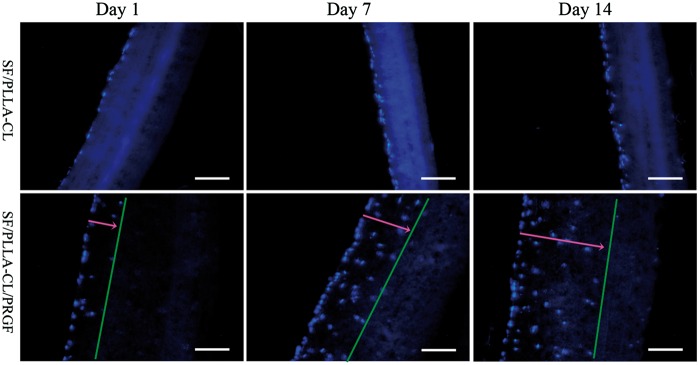
Fluorescence microscopy images of cell infiltration over a period of 1, 7 and 14 days. All images are displayed using a 200× magnification (scale bar = 100 μm)

HUASMCs were seeded on the grafts to study cellular infiltration. As demonstrated in Fig. 2, SF/PLLA-CL/PRGF significantly enhanced the cellular migration and infiltration into the graft. On the contrary, cells seeded on SF/PLLA-CL were confined to the graft surface and present no obvious infiltration. Cells could infiltrate deeper in SF/PLLA-CL/PRGF, partial because this kind of graft had a big pore size, which facilitated the cells migration. Moreover, grafts with PRGF-incorporation, the fibers deposited looser than that without PRGF as mentioned above (shown in Section Graft Characterization). It is suggested that the loosened fiber gave less resistance for cells to across, which resulted in a good cellular penetration.

The quantitative data for cellular infiltration was shown in Table 1. SF/PLLA-CL/PRGF caused the cellular infiltration with the average depth of 71 ± 24, 140 ± 10 and 247 ± 24 μm at 1, 7 and 14 days, respectively. According to the literature, in order to improve the cell infiltration into the electrospun graft, Baker et al. applied electrospun PCL intermingled with polyethylene oxide (PEO) fibrous graft, with the water-soluble PEO ‘sacrificial fibers’ removed by post-processing [32]. However, the cellular infiltration was depended on the expenses of a significant reduction in the grafts’ mechanical properties. In contrast, here SF/PLLA-CL graft with PRGF was fabricated, and the functional PRGF induced SMCs penetration into graft without any key mechanical properties changes, which would be shown in Section Mechanical Properties.

**Table 1.** Average depth results of cellular penetration

Specimen	Average depth of cellular penetration/(µm)		
	Day 1	Day 7	Day 14
SF/PLLA-CL	<10	<15	<20
SF/PLLA-CL/PRGF	71 ± 24	140 ± 10	247 ± 24

SMC-seeded grafts after 1, 7 and 14 days of *in vitro* culture.

#### HUASMCs proliferation

**Figure 3.**
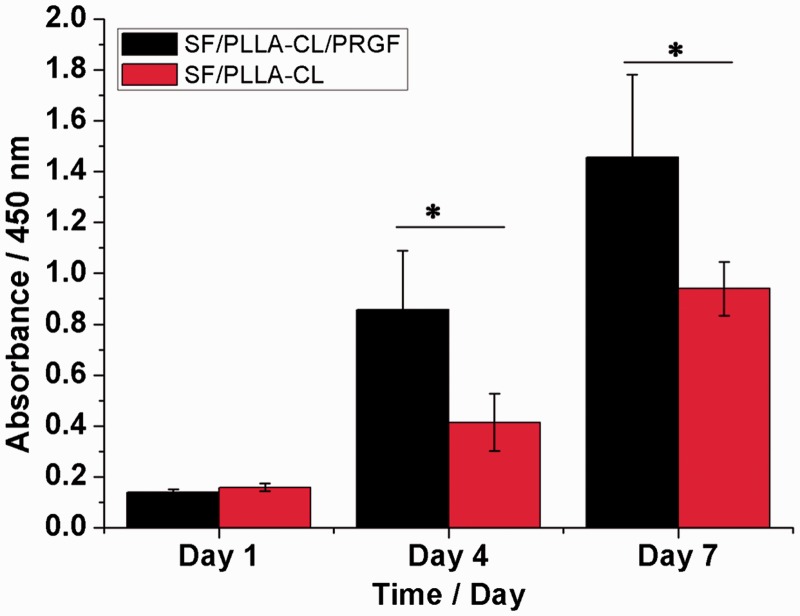
The proliferation of HUASMCs cultured on SF/PLLA-CL/PRGF and SF/PLLA-CL for 1, 4 and 7 days. Statistical difference between groups is indicated (*P < 0.05)

The proliferation of HUASMCs on days 1, 4 and 7 after seeding on the various grafts was shown in Fig. 3. As a whole, the SF/PLLA-CL/PRGF was conducive to HUASMC proliferation in comparison with SF/PLLA-CL. On day 4 and 7, HUASMC proliferation on SF/PLLA-CL/PRGF exhibited a significant increase (*P* < 0.05) compared to SF/PLLA-CL. The results showed that SF/PLLA-CL/PRGF could promote HUASMC growth and proliferation compared to SF/PLLA-CL. This was probably caused by the introduction of biological functional groups via PRGF in SF/PLLA-CL/PRGF that enhanced the proliferation rate of HUASMCs.

**Figure 4.**
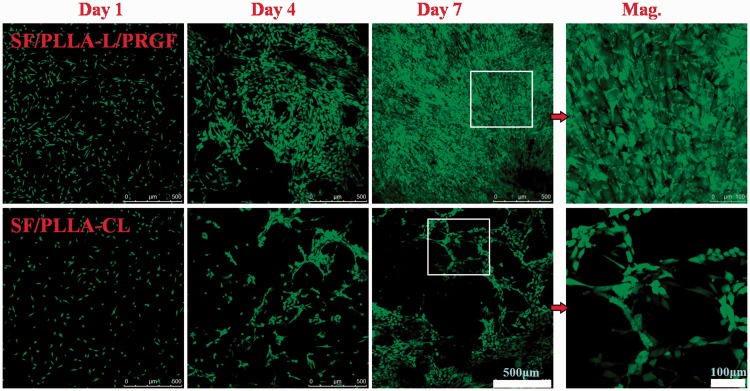
Confocal laser images of HUASMCs grown on SF/PLLA-CL/PRGF and SF/PLLA-CL for 1, 4 and 7 days

HUASMC morphology and the interaction between cells and grafts were studied *in vitro* for 1, 4 7 days. Confocal laser micrographs were shown in Fig. 4. Overall, HUASMCs had a fast growth rate, and the results were consistent with Fig. 3. After 4 days, the HUASMCs spread more easily to develop a SMC layer on the surface of SF/PLLA-CL/PRGF compared to the SF/PLLA-CL. Meanwhile, cells on SF/PLLA-CL/PRGF appeared as spindle-like shape and elongated along with the fiber aligned fluently especially on day 7, shown in the high-resolution of an area squared in day 7 (Mag.).

### Mechanical properties

Over the past 50+ years, different strategies for bridging the gap between synthetic materials and native vein and artery have been proposed. From early applications of ePTFE and Dacron to Bell’s landmark paper in 1986 which described the concept of a completely biological and cell-based graft produced *in vitro* [33], all of them have been hampered by poor mechanical strength especially in small diameter vascular application. Therefore, to fabricate a functional vascular graft, appropriate mechanical properties are required. Here, detailed mechanical data with tensile strength, suture retention, burst pressure and compliance for as-prepared grafts were shown.

#### Suture retention strength and tensile testing

Using electrospun fibers to form a non-woven fibrous scaffolding could enhance its porosity for use in vascular tissue engineering. However, it may raise concerns about its ability to retain suture retention upon implantation as a bioresorbable vascular prosthetic. Thus, the essential ability for suture retention was investigated (Table 2). The thickness for all samples was very close with a value at about 0.28 mm. The results showed that the suture retention strengths for both two samples were almost the same. SF/PLLA-CL/PRGF had an average value of 3.3 ± 0.2 N, and the SF/PLLA-CL had an average value of 3.4 ± 0.3 N. Both grafts possessed higher suture strength than that of native blood vessels, which was 1.7 N [34]. Therefore, the suture retention strength of both SF/PLLA-CL/PRGF grafts was adequate for clinical use during the surgical procedure.

**Table 2.** Mechanical properties of different grafts

Sample	Suture retention strength/*N*	Peak stress/MPa	Modulus/MPa	Strain at break/%
SF/PLLA-CL/PRGF	3.3 ± 0.2	4.1 ± 0.3	1.8 ± 0.2	282 ± 12
SF/PLLA-CL	3.4 ± 0.3	4.7 ± 1.6	2.5 ± 0.2	259 ± 23

Tensile tests were conducted to determine whether the properties were conducive for use as a vascular graft. The tensile testing results are shown in Table 2. As for SF/PLLA-CL/PRGF, the peak stress was 4.1 MPa compared to SF/PLLA-CL with a peak stress of 4.7 MPa. The stress for graft had a slight reduce with added PRGF. On the other hand, SF/PLLA-CL/PRGF possessed slightly higher strain (282%) and lower elastic modulus (1.8 MPa) than SF/PLLA-CL (259% and 2.5 MP, respectively). Overall, SF/PLLA-CL/PRGF had appropriate mechanical properties compared to native blood vessels, which had a strain of 45–99% [35], stress of 4.4 MPa [34], and elastic modulus of 2.68 ± 1.81 MPa [36].

#### Burst pressure and dynamic compliance

**Figure 5.**
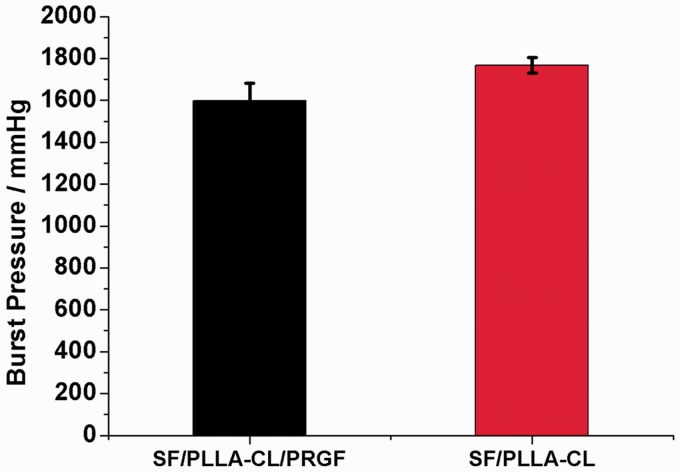
Burst pressure results of the P(LLA-CL)/SF/PRGF and SF/PLLA-CL grafts

The burst pressure of a vascular graft is one of the most important parameters which determine the suitability of its clinical use [37]. For this study, all the samples with an inner diameter of tubes at approximate 4 mm were soaked in PBS for 6 h before testing, and the thickness of specimens were measured (0.24–0.26 mm). The burst pressure results are presented in Fig. 5. It is showed that the SF/PLLA-CL/PRGF and SF/PLLA-CL had burst pressures of 1600 ± 80 and 1760 ± 37 mmHg, respectively. Although the burst pressure of SF/PLLA-CL/PRGF was a little lower than that of SF/PLLA-CL, it still presented equivalent value as saphenous vein which had the burst pressure values of 1599–2273 mmHg [38]. Therefore, the SF/PLLA-CL/PRGF graft could be used as an artery bypass graft.

**Figure 6.**
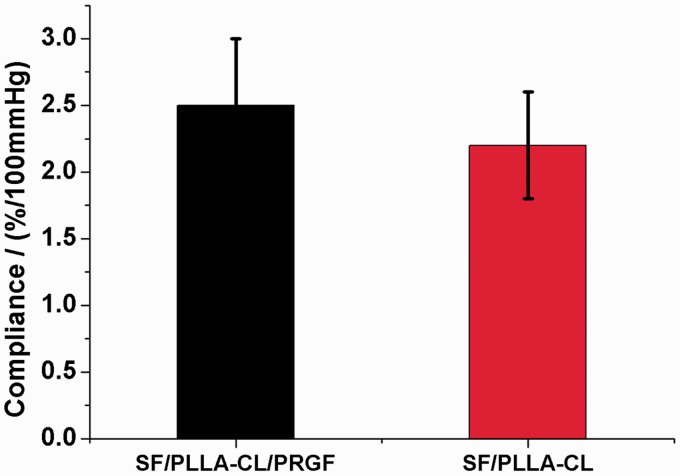
Compliance results of the P(LLA-CL)/SF/PRGF and SF/PLLA-CL grafts

Exhibiting adequate compliance that can match the native tissue for grafts is very important for the resistance to intimal hyperplasia. Dynamic compliance testing has been conducted, and the results are presented in Fig. 6. It showed that the compliance of the SF/PLLA-CL/PRGF and SF/PLLA-CL had an average compliance value of 2.5 ± 0.5, 2.2 ± 0.4%, respectively, with none of these grafts being significantly different from each other. Overall, the compliance values of the SF/PLLA-CL/PRGF approached that of native tissue (2.6%/100 mmHg) [39]. In addition, both grafts possessed higher compliance values than saphenous vein (0.7–1.5%/100 mmHg) [38], and standard ePTFE grafts (0.1%/100 mmHg) [40].

## Conclusion

This study demonstrated that the functional PRGF had an effect on the smooth muscle layer construction for electrospun SF/PLLA-CL polymer graft with PRGF. With the incorporation of PRGF into SF/PLLA-CL electrospun graft, SMCs could be promoted with fast growth. Moreover, PRGF enhanced cellular infiltration into the graft to a deep extent. Additionally, the SF/PLLA-CL/PRGF graft also possessed adequate mechanical properties in terms of tensile strength, suture retention strength, burst pressure and compliance which were comparable to that of native tissue. Thus, this type of graft shows great potential in applications such as biodegradable vascular substitutes.
